# Spinal mobilization force-time characteristics: A scoping literature review

**DOI:** 10.1371/journal.pone.0289462

**Published:** 2023-11-14

**Authors:** Lindsay M. Gorrell MChiroprac, Luana Nyirö, Mégane Pasquier, Isabelle Pagé, Nicola R. Heneghan, Petra Schweinhardt, Martin Descarreaux

**Affiliations:** 1 Department of Chiropractic Medicine, Integrative Spinal Research Group, University Hospital Balgrist and University of Zürich, Zürich, Switzerland; 2 Institut Franco-Européen de Chiropraxie, Toulouse, France; 3 Department of chiropractic, Université du Québec à Trois-Rivières, Trois-Rivières, QC, Canada; 4 Center for Interdisciplinary Research in Rehabilitation and Social Integration (Cirris), Centre Intégré Universitaire de Santé et de Services Sociaux de la Capitale-Nationale (CIUSSS-CN), Québec City, QC, Canada; 5 School of Sport, Exercise & Rehabilitation Sciences, University of Birmingham, Birmingham, United Kingdom; 6 Department of Human Kinetics, Université du Québec à Trois-Rivières, Trois-Rivières, QC, Canada; King Khalid University, SAUDI ARABIA

## Abstract

**Background:**

Spinal mobilization (SMob) is often included in the conservative management of spinal pain conditions as a recommended and effective treatment. While some studies quantify the biomechanical (kinetic) parameters of SMob, interpretation of findings is difficult due to poor reporting of methodological details. The aim of this study was to synthesise the literature describing force-time characteristics of manually applied SMob.

**Methods:**

This study is reported in accordance with the Preferred Reporting Items for Scoping Reviews (PRISMA-ScR) statement. Databases were searched from inception to October 2022: MEDLINE (Ovid), Embase, CINAHL, ICL, PEDro and Cochrane Library. Data were extracted and reported descriptively for the following domains: general study characteristics, number of and characteristics of individuals who delivered/received SMob, region treated, equipment used and force-time characteristics of SMob.

**Results:**

There were 7,607 records identified and of these, 36 (0.5%) were included in the analysis. SMob was delivered to the cervical spine in 13 (36.1%), the thoracic spine in 3 (8.3%) and the lumbopelvic spine in 18 (50.0%) studies. In 2 (5.6%) studies, spinal region was not specified. For SMob applied to all spinal regions, force-time characteristics were: peak force (0-128N); duration (10-120s); frequency (0.1–4.5Hz); and force amplitude (1-102N).

**Conclusions:**

This study reports considerable variability of the force-time characteristics of SMob. In studies reporting force-time characteristics, SMob was most frequently delivered to the lumbar and cervical spine of humans and most commonly peak force was reported. Future studies should focus on the detailed reporting of force-time characteristics to facilitate the investigation of clinical dose-response effects.

## Introduction

Musculoskeletal disorders, including low back and neck pain, affect most individuals during their lives [1–4]. Such disorders are a prominent cause of disability globally [5] and can lead to decreased quality of life and psychological distress [6,7]. The global prevalence of musculoskeletal disorders is increasing [8], as are their associated financial and societal costs [9–11]. Individuals commonly seek care from various healthcare providers for the treatment of musculoskeletal disorders [12–14] and the use of evidence-based interventions is recommended [12,15–17]. Clinical practice guidelines recommend the use of conservative treatments including spinal mobilization (SMob) and/or spinal manipulation (SM) for the treatment of musculoskeletal disorders [18–21]. SMob is characterized by the manual application of oscillatory forces with low velocity and variable amplitude and frequency to an articulation [22]. SMob can be further described in terms of the movement amplitude (or ’grade)’ as first described by Maitland [23]. Specifically: Grade I involves a small-amplitude movement performed at the beginning of the range of motion (ROM); Grade II is a large-amplitude movement performed within the free range but not moving into any resistance or stiffness; Grade III is a large-amplitude movement performed up to the limit of the range; and Grade IV involves a small-amplitude movement performed at the limit of the range. It has been reported that Grades I and II SMob are often applied with the intention of pain reduction, while Grades III and IV are commonly used to increase ROM [24].

Transient neurophysiological effects in both the autonomic (e.g. changes in skin temperature and conductance [25,26]) and somatic (e.g. changes in muscle activity [26,27]) nervous systems have been reported in response to SMob. Additionally, beneficial clinical outcomes such as hypoalgesia [25,28–30] and increased ROM [27,31] have been linked to the intervention. However, it is yet to be established if physiological responses to manual therapy (i.e. SMob) are related to clinical outcomes [32]. Therefore, the mechanisms underlying the beneficial clinical effects of SMob remain unclear [33] and without quantification of the intervention, it is difficult to determine which, if any, force-time characteristics may influence patient outcomes [34].

To date, there have been two reviews of SMob force-time characteristics [22,35] reporting on mean peak forces during SMob delivered in a posterior-anterior (PA) direction. In a 1997 review by Björnsdóttir and colleagues, force application was discussed in a single paragraph, with data reported for SMob delivered to: i) the L3 vertebra by 2 instructors (mean peak force: 33.3N); and ii) an unspecified thoracic level by 2 manual therapists using Grades I (means of the means for the 2 therapists: 134.75N) and IV (342.5N) [35]. In a 2006 review, Snodgrass and colleagues evaluated the literature for consistency of force application by manual therapists during PA SMob [22]. This review reported on mean peak forces in the PA direction for Grades I-IV SMob delivered to the spine (cervical:4; thoracic:3; and lumbar:7) and artificial devices (4). Both reviews highlighted a variability in nomenclature, definitions of force-time characteristics and force delivery during SMob [22,35]. Since the Snodgrass and colleagues review, there has been no further collation or synthesis of SMob force-time characteristics. Therefore, the aim of this study was to synthesise the existing literature describing biomechanical (kinetic) parameters during the delivery of manually applied SMob.

## Methodology

This scoping literature review is reported in accordance with the Preferred Reporting Items for Scoping Reviews (PRISMA-ScR) statement [36]. The protocol was developed by an experienced, international and interprofessional team and was prospectively registered at the Open Science Framework Registry (https://osf.io/3mqjs/). The original study design and subsequent search were conducted with the intention to capture information concerning the force-time characteristics of both SMob and SM. Protocol deviations included that: i) due to the large quantity of data published on the topic, it was decided to report the force-time characteristics of SMob and SM separately; and ii) studies reporting on SMob delivered to animals were excluded as it was unknown how biomechanically comparable SMob delivery would be to that delivered to humans. Due to the separate reporting of SMob and SM data in different manuscripts, several sections of the methods described here mirror those in the manuscript reporting on SM data [37].

### Eligibility criteria

Eligibility criteria were developed using the Sample, Phenomenon of Interest, Design, Evaluation, Research Type (SPIDER) search concept tool [38].

### Inclusion criteria

S–the sample population was humans (of any age) and inanimate objects (e.g. instrumented tool, manikin);

PI–the phenomenon of interest was manually delivered SMob, delivered by any regulated health professional (e.g. physiotherapist or chiropractor) or student enrolled at an accredited institution;

D–observational study designs (e.g. case series studies, cohort and case-control studies);

E–kinetic variables of the intervention (e.g. force-time profile); and

R–original quantitative research data from studies utilizing SMob as either the sole intervention or as a comparator.

### Exclusion criteria

Exclusion criteria were: i) studies that reported on SMob delivered by a mechanical instrument or device (e.g. Activator device or mechanical robot); ii) studies that reported on all other therapeutic modalities (e.g. soft tissue treatment, massage therapy); iii) manuscript not published in English, French or German; and iv) studies that had been retracted, were secondary analyses, trial registrations, protocols, clinical practice guidelines, commentaries, editorials, conference proceedings or single case studies.

### Search strategy

The search strategy was created by subject specific and methodological experts, with the assistance of an experienced medical and health sciences librarian. MEDLINE(Ovid), Embase, CINAHL, ICL, PEDro and Cochrane Library databases were searched from inception to 4 October 2022. The following search terms and derivatives were adapted for each search engine: (spine, spinal, manipulation, mobilization or mobilisation, musculoskeletal, chiropractic, osteopathy, physiotherapy, naprapathy, force, motor skill, biomechanics, dosage, dose-response, education, performance, psychomotor, back, neck, spine, thoracic, lumbar, pelvic, cervical, sacral). Search strategies for all databases are provided in S1 File.

### Study selection process

Records retrieved from the electronic searches were exported to the Rayyan© online platform (2022) [39] and duplicates were removed. Beginning with title and abstract review, groups of two authors (LG and LN; LG and IP; LG and MP) independently screened studies. Full-texts of the remaining studies were then retrieved and screened independently by groups of two authors (LG and LN; LG and IP). The first author (LG) screened the reference lists of included studies to examine whether all relevant literature was captured. No further studies were revealed by this process. Disagreements regarding study inclusion that could not be resolved by consensus were resolved by a third author (MD).

### Data extraction

Data were extracted from eligible studies by groups of two independent authors (LG and LN; LG and MP). These data included: i) general study characteristics (e.g. title, author, year and country of publication and type of study); ii) general study information (e.g. individual who delivered the intervention [e.g. clinician, student], professional qualification of individual delivering the intervention [e.g. physiotherapist, chiropractor], years of clinician experience/number of student hours, number of clinicians/students who delivered SMob, recipient [e.g. human, manikin], number of recipients, whether the intervention was SMob [and grade of mobilization], the region treated [e.g. cervical, thoracic] and the measurement equipment used to record force-time characteristics of the intervention [e.g. type and metrological details]); and iii) force-time characteristics of SMob (e.g. peak force, SMob duration and frequency and force amplitude).

### Definitions

In this study, the following definitions were used:

- Peak force: the maximum applied force during a single SMob, reported as the mean of the force peaks that occurred during a specified period of the intervention.
- Duration: the time period of SMob delivery.
- Frequency: the rate of force oscillation during repeated applications.
- Force amplitude: the difference between the minimum and maximum forces applied during the intervention (i.e. the difference between a peak force and trough), reported as the mean of the force amplitudes that occurred during a specified period of SMob.
- Metrological details: descriptions of the suitability (e.g. accuracy, precision, sensitivity) of the measurement equipment to quantify the force-time characteristics of SMob [40].

### Data synthesis

Descriptive statistics (mean, standard deviation and range) were used to report data. Any deviations from this (such as the use of 95% confidence intervals or the reporting of median and interquartile range) are explicitly indicated and reflect how the data were reported in the original studies. Microsoft Excel (Office 365, Microsoft Corporation, Redmond, USA) was used to calculate frequencies and proportions of trials reporting on each of the specified domains mentioned above.

In order to manage the substantial volume of data presented in this study, the following decisions were made regarding how to best report the data:: i) for studies reporting forces measured in 3-dimensions (3D) and including the resultant forces (i.e. the total forces applied), only the resultant forces are reported; ii) for studies measuring forces applied in 3D but not including the resultant forces, only the forces measured in the primary direction of the applied force are reported in the tables (e.g. for prone PA thoracic SMob, the vertical forces are reported). Regarding the reporting of metrological data, a consensus was reached by two authors (LG and LN; LG and MP). The final decision as to whether adequate information was provided was reached by consensus by LG and MD. In cases where metrological details were discussed (e.g. it was stated that measurement equipment accuracy was good) but it was not clear if this statement was based on data (or what data), this was recorded as metrological details were not provided. No assessment of study quality was performed.

## Results

**Fig 1:**
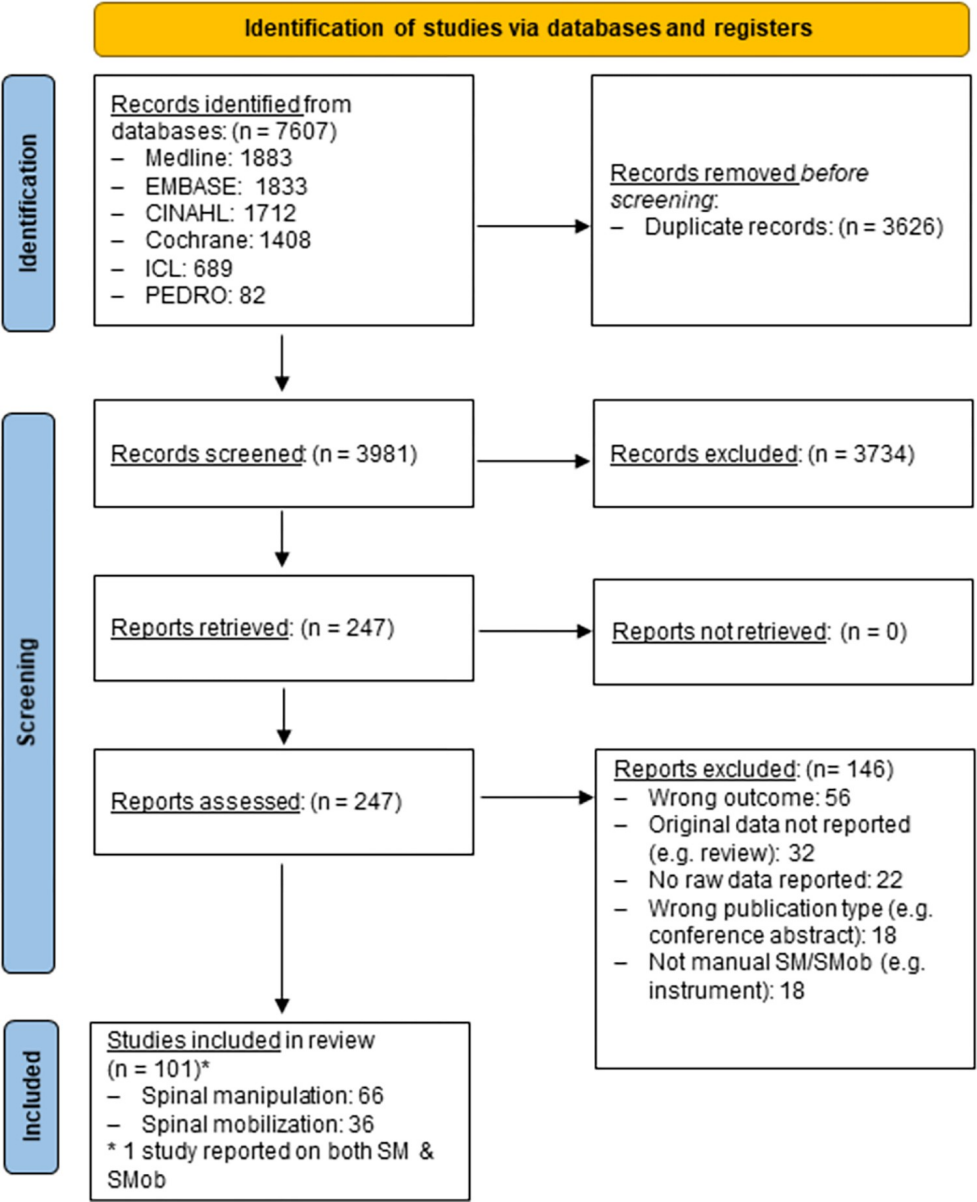
PRISMA flow-chart.

The electronic searches returned 7,607 records, with 3,981 unique records remaining after de-duplication (n = 3,626) (Fig 1). Following title/abstract screening, 247 full-texts were screened and146 reports were excluded (e.g. did not report force-time characteristics: 56), leaving 101 included studies of which 36 reported on SMob and were included in the analysis. A list of these studies is provided in S2 File and the reference number cited in the tables refers to this list.

Most of the 36 included studies were published in the 10-year period from 2003 to 2012 (n = 13, 36.1%) in Australia (n = 13, 36.1%) (Table 1). Typically, the study design was cross-sectional (n = 26, 72.2%), with SMob delivered by a clinician only (i.e. no students were involved) (n = 24, 66.7%) whose profession was a physiotherapist (n = 27, 75.0%). In the 31 (86.1%) studies in which SMob was delivered by clinicians, clinical experience was unclear in 14 (45.2%) studies and when it was reported, clinicians with more than 5 years’ experience most commonly delivered SMob (n = 10, 32.3%). When SMob was delivered by a student (n = 10, 27.8%), the number of SMob training hours was not reported in any (n = 10, 100.0%) study. Most frequently, the number of individuals (i.e. clinicians and/or students) delivering SMob was between 1 and 49 (n = 29, 80.6%), with 12 (33.3%) of these studies involving only 1 to 2 individuals delivering SMob. SMob was delivered to adults (18 to 65 years) in 20 (55.6%) studies, with the demographics of the cohort to which SMob was delivered not reported in 8 (22.2%) studies. The number of individuals receiving SMob was reported as between 1 and 49 in 28 (77.8%) studies, with only 1 to 2 individuals receiving SMob in 10 (27.8%) studies. SMob was most commonly delivered to the lumbopelvic spine (n = 18, 50.0%) and the cervical spine (n = 13, 36.1%), and the SMob ’technique’ was reported in all but one study (n = 35, 97.2%). Force-time characteristics were measured at the patient-table interface in 16 (44.4%) studies, another interface (e.g. thumbnail of the clinician) in 6 (16.7%) studies, the clinician-patient interface in 5 (13.9) studies and the clinician-ground interface in 4 (11.1%) studies. Regarding force-time characteristics, the following were reported: peak force in 35 (97.2%); SMob duration in 12 (33.3%); SMob frequency in 16 (44.4%); and amplitude of force in 11 (30.6%) studies. Metrological data of the measurement equipment were reported in 27 (75.0%) studies.

**Table 1:** Overall summary of studies reporting on the force-time characteristics of spinal mobilization (SMob) (n = 36).

	n, (%)		n, (%)
Year, n = 36		Individual who received SMob, n = 36	
2013 to 2022	12 (33.3)	Adult (18-65yr)	20 (55.6)
2003 to 2012	13 (36.1)	Geriatric (>65yr)	1 (2.8)
1993 to 2002	10 (27.8)	Instrumented tool/force plate	4 (11.1)
Before 1993	1 (2.8)	Manikin	1 (2.8)
Country, n = 36		Mixed	2 (5.6)
Australia	13 (36.1)	Unclear	8 (22.2)
Canada	6 (16.7)	Number of individuals receiving SMob, n = 36	
England	6 (16.7)	1 or 2	10 (27.8)
Ireland	1 (2.8)	0 to 49	28 (77.8)
Malaysia	1 (2.8)	50 to 99	5 (13.9)
South Africa	1 (2.8)	Not reported	3 (8.3)
Unclear	1 (2.8)	Region SMob delivered to, n = 36	
USA	7 (19.4)	Cervical	13 (36.1)
Study type, n = 36		Thoracic	3 (8.3)
Cross-sectional	26 (72.2)	Lumbopelvic	18 (50.0)
Prospective	10 (27.8)	Other	2 (5.6)
Individual who delivered SMob, n = 36		Technique reported, n = 36	
Practitioner	24 (66.7)	Yes	35 (97.2)
Student	5 (13.9)	No	1 (2.8)
Both	5 (13.9)	Measurement interface, n = 36	
Unclear	2 (5.6)	Patient-table	16 (44.4)
Profession, n = 36		Clinician-patient	5 (13.9)
Physiotherapist	27 (75.0)	Clinician-ground	4 (11.1)
Chiropractor	5 (13.9)	Table-ground	3 (8.3)
Unclear	4 (11.1)	Both clinican-patient & patient-table	1 (2.8)
Experience (clinician) n = 31		Other	6 (16.7)
>5yr	10 (32.3)	Unclear	3 (8.3)
Mixed	7 (22.6)	Metrological data reported, n = 36	
Unclear	14 (45.2)	Reported	27 (75.0)
Hours of training (student) n = 12		Not reported	9 (25.0)
Reported	0 (0.0)	Peak force, n = 36	
Not reported	12 (100.0)	Reported	35 (97.2)
Number of individuals delivering SMob, n = 36		Not reported	1 (2.8)
1 or 2	12 (33.3)	Duration of mobilization, n = 36	
1 to 49	29 (80.6)	Reported	12 (33.3)
50 to 99	2 (5.6)	Not reported	24 (66.7)
100 to 149	2 (5.6)	Frequency of mobilization, n = 36	
>150	1 (2.8)	Reported	16 (44.4)
Not reported	2 (5.6)	Not reported	20 (55.6)
		Amplitude of force, n = 36	
		Reported	11 (30.6)
		Not reported	25 (69.4)

Abbreviations: n: Number of studies, SMob: Spinal mobilization, y: Year, >: Greater than, <: Less than.

### Cervical spine

Thirteen studies (36.1%) reported on SMob delivered to the cervical spine. Eleven (84.6%) of these studies reported on SMob delivered to the cervical spine of humans and the following force-time characteristics were reported: i) peak force: 0-128N; ii) duration: 60s; iii) frequency: 0.28–2.4Hz; and iv) force amplitude: 14.4–52.5N (detailed information provided in: Tables 2 & 3; S3 Appendix, Table A in S3 File). Two (15.4%) of these studies reported on SMob delivered to inanimate objects (i.e. human analogue manikin:1; instrumented tool:1) and peak forces 42-181N were reported.

**Table 2:** Summary of force-time characteristics reported by region for studies reporting on spinal mobilization (SMob) (n = 36).

	Location of measurement n (%)	Metrologic data reported n (%)	Peak force reported n (%) [range (N)]	Duration reported n (%) [range (s)]	Frequency reported n (%) [range (Hz)]	Force amplitude reported n (%) [range (N)]
**Cervical spine**						
Humans (n = 11)	Patient-table: 8 (72.7) Clinician-patient: 1 (9.1) NR: 1 (9.1) Other: 1 (9.1)	3 (27.3)	11 (100.0) [0–128]	1 (9.1) 60	6 (54.5) [0.3–2.4]	4 (36.4) [14.4–52.5]
Inanimate objects (n = 2)	Within device: 1 (50.0) Clinician-device: 1 (50.0)	0 (0.0)	2 (100.0) [42–181]	0 (0.0) [NA]	0 (0.0) [NA]	0 (0.0) [NA]
**Thoracic spine**						
Humans (n = 2)	Clinician-patient: 1 (50.0) Clinician-patient & patient-table: 1 (50.0)	1 (50.0)	1 (50.0) [297–323]	1 (50.0) 3x60	1 (50.0) [28–32^Δ^]	0 (0.0) [NA]
Inanimate objects (n = 1)	Clinician-device & table-ground: 1 (100.0)	0 (0.0)	1 (100.0) [106–223]	0 (0.0) [NA]	1 (100.0) 0.5	0 (0.0) [NA]
**Lumbar spine**						
Humans (n = 17)	Patient-table: 7 (41.2) Clinician- ground: 4 (23.5) Table-ground: 3 (17.6) Clinician-patient: 2 (11.8) NR: 1 (5.9)	3 (17.6)	16 (94.1) [3–430]	8 (47.1) [10–120]	7 (41.2) [0.1–4.5]	7 (41.2) [1–102]
Inanimate objects (n = 1)	Device-table: 1 (100.0)	0 (0.0)	1 (100.0) [36–119]	1 (100.0) 30	0 (0.0) [NA]	0 (0.0) [NA]
**No region specified**						
Inanimate objects (n = 2)	Within device: 1 (50.0) Clinician-device & Clinician-ground: 1 (50.0)	0 (0.0)	2 (100.0) [2–361]	1 (50.0) 20	1 (50.0) [28–32^Δ^]	NR

Abbreviations: Hz: Hertz, N: Newtons, n: Number of studies, NA: Not applicable, NR: Not reported, Other: Clinician thumb nail bed, s: Seconds, SMob: Spinal mobilization, Δ: Data are reported as cycles/minute.

**Table 3:** Summary of studies reporting on the force-time characteristics of spinal mobilization (SMob) delivered to the cervical spine of humans (n = 11) and inanimate objects (i.e. human analogue manikins, instrumented tools) (n = 2).

Author/s Year, Country	SMob delivery Profession (n)	Experience	Recipient/s (n)	Location/s	Technique/s Grade/s	Interface/s	Equipment	Metrological data
**Humans**								
Conradie et al 2004, South Africa [7]	Clin Physio (16)	NR	Adult (1)	C6	PA I	Clin-pat	Pressure sensor	No
Snodgrass et al 2006, Australia [28]	Clin Physio (10)	Mixed	Adult (1)	C2/C7	PA I-IV	Pat-table	Load cells	No
Snodgrass et al 2009, Australia [29]	Clin Physio (116)	>5y	Adult (35)	C2/C7	PA I-IV	Pat-table	Load cells	Yes
Snodgrass et al 2010, Australia [30]^Ʊ^	Clin & Stud Physio (336)	Clin: NR Stud: NR (no clin exper)	Adult (67)	C2/C7	PA I-IV	Pat-table	Load cells	Yes
Snodgrass et al 2010, Australia [31]	Stud Physio (120)	NR (2–4 y)	Adult (32)	C2/C7	PA I-IV	Pat-table	Load cells	Yes
Gudavalli et al 2013, USA [13]	Clin Chiro (4)	NR	Adult (9)	C5/C6	C distract NA	Pat-table	Force plate	No
Snodgrass et al 2014, Australia [33]	Clin Physio (1)	>5y	Adult (64)	MP (C3-7)	PA III	Pat-table	Load cells	No
Gudavalli et al 2015, USA [15]	Clin Chiro (NR)	NR	Adult (45)	Occi/C5	C distract NA	Pat-table	Force plate	No
Gudavalli et al 2015, USA [16]	Clin Chiro (2)	>5y	Adult (48)	Occi/C5	C distract NA	Pat-table	Force plate	No
Kope et al 2018, Canada [19]	Clin Physio (5)	>5y	NR (NR)	C5/C6	PA III	Clin thumbnail	Strain gauge	No
Chia et al 2021, Malaysia [4]	Clin Physio (1)	NR	NR (30)	C6	PA I-IV	NR	Pressure sensor	No
**Inanimate objects**								
Buckingham et al 2007, Australia [3]	Clin & Stud Physio (36)	Clin: Mixed Stud: NR (4^th^ y)	Manikin (1)	C6	PA NR	In manikin	Strain gauge	No
Walsh et al 2011, Ireland [35]	Stud Physio (40)	NR (final y)	Instrumented tool (1)	C7	PA III	Instrumented tool	Pinch-grip analyser	No

Abbreviations: C: Cervical, Chiro: Chiropractor, Clin: Clinician, distract: Distraction, exper: Experience, Mixed: Experience of clinicians both > and < 5 years, MP: Most painful, (n): Number of participants, NA: Not applicable, NR: Not reported, Occi: Occiput, PA: Posterior-anterior, Pat: Patient, Physio: Physiotherapist, SMob: Spinal mobilization, Stud: Students, y: Years, >: Greater than.

### Thoracic spine

Three studies (8.3%) reported on SMob delivered to the thoracic spine. Two (66.7%) of these studies reported on SMob delivered to the thoracic spine of humans and the following force-time characteristics were reported: i) peak force: 297-323N; ii) duration: 3x60s; and iii) frequency: 0.47–0.53Hz (detailed information provided in: Tables 2 & 4; S3 Appendix, Table B in S3 File). In the one (33.3%) study that reported on SMob delivered to 12 T5-8 sections of human cadavers: i) peak force: 106-223N; and ii) frequency: 0.5Hz were reported.

**Table 4:** Summary of studies reporting on the force-time characteristics of spinal mobilization (SMob) delivered to the thoracic spine of humans (n = 2) and inanimate objects (i.e. partial cadaveric sections) (n = 1).

Author/s Year, Country	SMob delivery Profession (n)	Experience	Recipient/s (n)	Location/s	Technique/s Grade/s	Interface/s	Equipment	Metrological data
**Humans**								
Zegarra-Parodi et al 2016, NR [36]	NR NR (1)	NR	Adult (32)	T1	Lateral glide via lamina NR (5/40/80% of PPT)	Clin-pat	Pressure sensor	No
Funabashi et al 2021, Canada [10]	Clin Chiro (1)	>5y	Geriatric (18)	T1-12	Clin choice 4	Clin-pat Pat-table	Load cells Force plate	Yes
**Inanimate objects**								
Sran et al 2004, Canada [34]	Clin Physio (2)	NR	Cadaveric sections T5-8 (12)	T6	PA NR	Clin-pat Table-floor	Pressure sensor	No

Abbreviations: Chiro: Chiropractor, Clin: Clinician, (n): Number of participants, NR: Not reported, PA: Posterior-anterior, Pat: Patient, Physio: Physiotherapist, PPT: Pressure pain threshold, SMob: Spinal mobilization, T: Thoracic, y: Years, >: Greater than.

### Lumbopelvic spine

Eighteen studies (50.0%) reported on SMob delivered to the lumbar spine. Seventeen (94.4%) of these studies reported on SMob delivered to the lumbar spine of humans and the following force-time characteristics were reported: i) peak force: 3-430N; ii) duration: 10-120s; iii) frequency: 0-5Hz; and iv) force amplitude: 1-102N (detailed information provided in: Tables 2 & 5; S3 Appendix, Table C in S3 File). In the one (33.3%) study that reported on SMob delivered to an instrumented tool: i) peak force: 36-119N; and ii) duration: 30s were reported.

**Table 5:** Summary of studies reporting on the force-time characteristics of spinal mobilization (SMob) delivered to the lumbopelvic spine of humans (n = 17) and inanimate objects (i.e. instrumented tools) (n = 1).

Author/s Year, Country	SMob delivery Profession (n)	Experience	Recipient/s (n)	Location/s	Technique/s Grade/s	Interface/s	Equipment	Metrological data
**Humans**								
Lee et al 1990, Australia [21]	Stud Physio (53)	NR (3^rd^ y)	NR (1)	L3	PA II	Table-ground	Force plate	No
Petty 1995, England [23]	Clin Physio (1)	NR	Adult (18)	L3	PA IV	Clin-ground	Force plate	No
Harms & Bader 1997, England [17]	Clin Therapist (30)	NR	Adult (1)	L3	PA I-IV/endfeel	Pat-table	Load cells	No
Harms et al 1999, England [18]	Clin Therapist (1)	<5y	Adult (61)	L3	PA I-IV	Pat-table	Load cells	No
Goodsell et al 2000, Australia [12]	Clin Physio (1)	NR	Mixed ages (26)	MP	PA NR	Pat-table	Load cells	No
Allison et al 2001, Australia [1]	Clin Physio (1)	NR	Adult (24)	L3	PA NR	NR	Force transducer	No
Chiradejnant et al 2001, Australia [5]	Clin Physio (3)	NR	NR (3)	L3	PA II/IV	Pat-table	Load cells	No
Chiradejnant et al 2002, Australia [6]	Clin Physio (10)	NR	Mixed ages (80)	L1-5	PA I-IV	Pat-table	Load cells	Yes
Cook et al 2002, USA [8]	Clin Physio (23)	Mixed	Adult (2)	L3	PA I-IV	Clin-ground	Force plate	No
Cook 2003, USA [9]	Clin Physio (22)	Mixed	NR (2)	L3	PA I-IV	Clin-ground	Force plate	No
Krekoukias et al 2009, England [20]	NR Therapist (1)	NR	Adult (36)	L3	PA NR	Clin-ground	Force plate	No
Sheaves et al 2011, Australia [25]	Stud Physio (62)	NR (3^rd^ y)	NR (62)	L3	PA II	Pat-table	NR	Yes
Snodgrass & Odelli 2012, Australia [32]	Clin & Stud Physio (27)	Clin: >5y Stud: NR	NR (26)	L3	PA I-IV	Pat-table	Load cells	Yes
Shum et al 2013, England [26]	Clin Physio (1)	>5y	Adult (39)	L4	PA III	Table-ground	Force plate	No
Gudavalli & Cox 2014, USA [14]	Clin Chiro (10)	Mixed	Adult (4)	NR	Cox FD NA	Clin-pat	Force transducer	No
Gagnon et al 2016, Canada [11]	Clin & Stud Physio (8)	Clin: >5y Stud: NR (4^th^ y)	Adult (5)	L2/L4	PA I-IV	Table-ground	Force plate	No
Petersen et al 2020, USA [22]	Stud Physio (24)	NR (1^st^ y)	Adult (24)	L3	PA III/IV	Clin-pat	Pressure sensor	No
**Inanimate objects**								
Björnsdóttir & Kumar 2003, Canada [2]	Clin & Stud Physio (20)	Clin : >5y Stud : NR (< 1y grad)	Instrumented tool (1)	NR	PA II	Pat-table	Load cell	No

Abbreviations: Chiro: Chiropractor, Clin: Clinician, FD: Flexion-distraction, Grad: Graduation, L: Lumbar, Mixed: Experience of clinicians both > and < 5 years, MP: Most painful level, (n): Number of participants, NR: Not reported, PA: Posterior-anterior, Pat: Patient, Physio: Physiotherapist, Stud: Students, SMob: Spinal mobilization, y: Years, >: Greater than, <: Less than.

### No region specified

Two (5.6%) studies reported on SMob delivered to an unspecified region and the following force-time characteristics were reported: i) peak force: 2-361N; ii) duration: 20s; and iii) frequency: 28–32 cycles/min (detailed information provided in: Tables 2 & 6; S3 Appendix, Table D in S3 File).

**Table 6:** Summary of studies reporting on the force-time characteristics of spinal mobilization (SMob) delivered to inanimate objects (i.e. instrumented tools) with no region specified (n = 2).

Author/s Year, Country	SMob delivery Profession (n)	Experience	Recipient/s (n)	Location/s	Technique/s Grade/s	Interface/s	Equipment	Metrological data
**Inanimate objects**								
Simmonds et al 1995, Canada [27]	Clin Physio (10)	>5y	Instrumented tool (1)	NA	PA I-V	In tool	Pinch-grip analyser	No
Petty & Messenger 1996, England [24]	Clin Physio (1)	NR	Instrumented tool (1)	NA	PA NR	Clin-tool Clin-ground	Pinch-grip analyser Force platform	No

Abbreviations: Clin: Clinician, (n): Number of participants, NA: Not applicable, NR: Not reported, PA: Posterior-anterior, Physio: Physiotherapist, SMob: Spinal mobilization, y: Years, >: Greater than.

## Discussion

This scoping review comprehensively synthesizes the existing evidence describing force-time characteristics during the delivery of manually applied SMob, underscoring the substantial variability observed in these parameters. This finding is consistent with the results of two previous reviews reporting on SMob peak forces delivered in a PA direction [22,35], despite the current study having a larger scope of reporting (i.e. duration, frequency and force amplitude in addition to peak forces). The observed heterogeneity in the reported force-time characteristics of SMob is likely attributable to several factors, such as differences in the: i) ’technique’ used (e.g. PA vs oscillatory distractive techniques); ii) measurement equipment used (e.g. force plate vs. pressure pad); iii) location of force-time characteristic measurement (e.g. clinician-patient vs. patient-table); iv) individuals/equipment to which SMob was applied (e.g. body mass index (BMI), equipment materials); v) terminology used to define SMob force-time characteristics and thus used to perform calculations (e.g. was the peak force measured directly by the equipment or, was it mathematically estimated?); and vi) individuals who delivered SMob (e.g. experience). However, without detailed descriptions of each of these domains, it is not possible to know if the identified force-time characteristic variability is related to methodological and/or reporting differences, inherent differences in SMob application or, some other factor [41]. Additionally, with the continued increase in the reporting on SMob force-time characteristic data, it is imperative that detailed descriptions are reported in manuscripts to allow readers the opportunity to assess for themselves possible reasons for differences in the data.

### Detailed reporting of the intervention is necessary

It is unknown if meaningful clinical differences between different types of SMob ’technique’ (e.g. PA vs. oscillatory distraction) and/or grades of SMob exist [34]. To assess this in future studies, there should be a detailed description of the applied SMob, including an explicit explanation of how the grade of SMob was defined. In the current review, detailed descriptions of SMob were not consistently provided. Specifically, there was large variation in the detail of SMob reporting and in many studies, replication of the intervention would be impossible based on the (lack of) details provided. Despite the existence of established reporting guidelines for health-related interventions [41], this lack of detail is not unique to SMob, existing also for interventions including SM [37] and dry needling [42]. It is recommended that a specific guideline for the standardized reporting of SMob, similar to that for the standardized reporting of SM [43], is developed to improve the reporting of SMob interventions. Indeed the (development and) use of such a guideline would go some way to improving the generally poor level of manual therapy trials reporting [44].

It is not yet fully understood why forces measured at the clinician-patient and patient-table interfaces differ [45,46]. However, as differences do exist, it is important for authors to report where and how force-time characteristics were measured as results from studies measuring forces at different interfaces are not directly comparable. Furthermore, the terminology used should enable replication of study methodologies in future investigations and would be facilitated by the detailed reporting of force-time characteristic definitions and calculations (e.g. was the peak force measured directly by the equipment or, was it mathematically estimated?). Without such details, it is impossible to compare force-time characteristics between studies. Furthermore, it has been reported that demographics (e.g. sex, height and BMI) influence the application of manual therapy treatments (i.e. SMob and SM) [47–50]. Specifically, these interventions are delivered more forcefully to males with a higher BMI. In summary, the interface of measurement, equipment used, terminology and SMob recipients should be systematically reported in detail to allow for both replication in future studies and reader judgement of the clinical relevance of reported results.

### Do force-time characteristics matter clinically?

It has been reported that force-time characteristics of SMob (e.g. peak force and force amplitude) differ between students and experienced clinicians [48]. Specifically, students apply less force, more slowly. Similar differences are also reported in the SM literature [51], further suggesting that the detailed reporting of the clinical experience of the individual delivering the intervention is necessary. Furthermore, Gorgos and colleagues reported on the reliability of inter-clinician and intra-clinician forces applied during joint mobilisation in a systematic review [52]. The authors concluded that while there is variability in the application of force between different clinicians, individual clinicians apply forces consistently. Despite such between-clinician differences in SMob force application, the literature shows that recipients experience beneficial clinical outcomes from various forms of manual therapy (including SMob) notwithstanding considerable differences in the force-time characteristics of the applied interventions (e.g. low velocity, variable amplitude SMob vs. high velocity, low amplitude SM) [34,53,54]. Additionally, some authors suggest that there is a threshold of ’dosage’ (in terms of force-time characteristics such as force and/or rate of force application), rather than an optimal intervention approach (i.e. SMob vs SM), required to elicit beneficial clinical outcomes [55,56]. However, to our best knowledge, this subject has not been systematically investigated and no reference standards (i.e. ranges of force-time characteristics) for SMob application have been published [52]. Furthermore, the lack of detailed description of force-time characteristics limits the generalizability of results reported by both individual studies, and their subsequent syntheses, to clinical practice as it remains unclear exactly what ’dosage’ was applied [42]. By exhaustively collating the existing literature, the current review provides a first step towards the development of such reference standards. However, the systematic biomechanical quantification of SMob is required to first establish if ’dosage’ is related to physiological responses (e.g. changes in the autonomic and somatic nervous systems) and/or clinical outcomes (e.g. hypoalgesia).

### Strengths and limitations

This review is the first to synthesise SMob force-time characteristic data beyond peak force (including also duration, frequency and force amplitude) and includes 21 additional studies since the publication of the most recent 2006 review [22]. The review was conducted by an international and interprofessional team and reported according to the (PRISMA-ScR) statement [36]. The study provides a first step towards the systematic and detailed reporting of SMob interventions, which is necessary to investigate the relationship between the application of SMob and its’ observed clinical outcomes.

It is possible that there was unintentional exclusion of studies reporting on the parameters of interest. However, it is unlikely that seminal studies were excluded for several reasons: i) a comprehensive search strategy was developed by an international, interprofessional team with relevant methodological and clinical expertise with the assistance of an experienced medical sciences librarian; ii) the search strategy was piloted and refined prior to being used; and iii) the review was conducted in a systematic fashion (i.e. using groups of two independent reviewers and data extractors). While it was intended that only original quantitative research data from studies utilizing SMob would be reported, it was not always clear as to whether reported data were previously published in part or fully. However, in instances where this was unclear, the decision was made to include the data. This decision ensured that the current review reported exhaustively on all studies reporting force-time characteristics of SMob. It is recommended that secondary analyses of data are transparently reported as such [57,58], with citation of the original publication, allowing readers to identify that the data has been previously published and to interpret for themselves the impact of the re-reported data. Finally, due to the considerable heterogeneity within studies reporting on force-time characteristics of SMob, no critical appraisal of the included studies was performed. However, it has been reported that there is currently no evidence to suggest that a lack of critical appraisal of the included studies impacts on the update and relevance of results reported in scoping reviews [59].

## Conclusion

This study has, as a first step, synthesised the current state of manually applied SMob force-time characteristic reporting. Most studies reported on SMob delivered to human lumbar or cervical spines, with peak force the most commonly reported parameter. Other reported parameters included duration, frequency and force amplitude. These findings highlight that considerable variability exists in the literature regarding SMob force-time characteristics. Future studies should focus on the detailed reporting of force-time characteristics which may facilitate the systematic investigation of dose-response effects clinically and the future development of reference standards (e.g. ranges of forces) for optimal intervention delivery.

## Supporting information

S1 Checklist Preferred Reporting Items for Systematic reviews and Meta-Analyses extension for Scoping Reviews (PRISMA-ScR) checklist. (DOCX)

S1 File Search strategies. (DOCX)

S2 File Included studies reference list. (DOCX)

S3 File Full results tables. (DOCX)
