# Supplementary material for: Spinal mobilization force-time characteristics: A scoping literature review
Source: PLoS One. 2023 Nov 14;18(11):e0289462. doi: 10.1371/journal.pone.0289462 (PMC10645339; doi:10.1371/journal.pone.0289462)
Supplement: S2 File — (DOCX) [file pone.0289462.s003.docx]

1. Allison G, Edmonston S, Kiviniemi K, Lanigan H, Simonsen AV, Walcher S. Influence of standardized mobilization on the posteroanterior stiffness of the lumbar spine in asymptomatic subjects. Physiother Res Int. 2001;6(3):145–56.

2. Björnsdóttir SV, Kumar S. Posteroanterior motion test of a lumbar vertebra: Accurcy of perception. Disabil Rehabil. 2003;25(4–5):170–8.

3. Buckingham G, Das R, Trott P. Position of undergraduate students’ thumbs during mobilisation is poor: an observational study. Aust J Physiother. 2007;53(1):55–9.

4. Chia KP, Li OK, Yuong TS, Singh OP, Faudzi A, Sornambikai S, et al. New real-time force monitoring device with enhanced accuracy for evaluation of applied force during the joint mobilization technique. Technol Health Care. 2021;29(4):829–36.

5. Chiradejnant A, Maher CG, Latimer J. Development of an instrumented couch to measure forces during manual physiotherapy treatment. Man Ther. 2001 Nov;6(4):229–34.

6. Chiradejnant A, Latimer J, Maher CG. Forces applied during manual therapy to patients with low back pain. J Manipulative Physiol Ther. 2002 Jul;25(6):362–9.

7. Conradie M, Smit E, Louw M, Prinsloo M, Loubser L, Wilsdorf A. Do experienced physiotherapists apply equal magnitude of force during a grade I central PA on the cervical spine? S Afr J Physiother. 2004;60(4):18–35.

8. Cook C, Turney L, Ramirez L, Miles A, Haas S, Karakostas T. Predictive factors in poor inter-rater reliability among physical therapists. J Man Manip Ther. 2002;10(4):200–5.

9. Cook CE. Effectiveness of visual perceptual learning on inter-therapist reliability of lumbar spine mobilization. Internet J Allied Health Sci Pract. 2003;1(2):11p–11p.

10. Funabashi M, Son J, Pecora CG, Tran S, Lee J, Howarth SJ, et al. Characterization of thoracic spinal manipulation and mobilization forces in older adults. Clin Biomech. 2021 Aug 14;89:105450.

11. Gagnon DH, Longtin C, Berbiche D, Gaudreault N. Do experienced physiotherapists and final year physiotherapy trainees apply similar force during posterior-to-anterior lumbar mobilization techniques? Man Ther. 2016 Feb;21:287–91.

12. Goodsell M, Lee M, Latimer J. Short-term effects of lumbar posteroanterior mobilization in individuals with low-back pain. J Manipulative Physiol Ther. 2000 Jun;23(5):332–42.

13. Gudavalli MR, Potluri T, Carandang G, Havey RM, Voronov LI, Cox JM, et al. Intradiscal Pressure Changes during Manual Cervical Distraction: A Cadaveric Study. Evid Based Complement Alternat Med. 2013;2013:954134.

14. Gudavalli MR, Cox JM. Real-time force feedback during flexion-distraction procedure for low back pain: A pilot study. J Can Chiropractic Assoc. 2014 Jun;58(2):193–200.

15. Gudavalli MR, Salsbury SA, Vining RD, Long CR, Corber L, Patwardhan AG, et al. Development of an attention-touch control for manual cervical distraction: a pilot randomized clinical trial for patients with neck pain. Trials. 2015 Jun;16:259.

16. Gudavalli MR, Vining RD, Salsbury SA, Corber LG, Long CR, Patwardhan AG, et al. Clinician proficiency in delivering manual treatment for neck pain within specified force ranges. Spine J. 2015 Apr;15(4):570–6.

17. Harms MC, Bader DL. Variability of forces applied by experienced therapists during spinal mobilization. Clin Biomech. 1997 Sep;12(6):393–9.

18. Harms MC, Innes SM, Bader DL. Forces measured during spinal manipulative procedures in two age groups. Rheumatology (Oxford). 1999 Mar;38(3):267–74.

19. Kope R, O’Brien J, Sadi J, Walton DM, Ferreira LM. Quantifying performance metrics of cervical spine mobilization for improved education and clinical outcomes: Early experience with a novel wearable device. J Rehabil Assist Technol Eng. 2018 Jan;5:2055668318765396.

20. Krekoukias G, Petty NJ, Cheek L. Comparison of surface electromyographic activity of erector spinae before and after the application of central posteroanterior mobilisation on the lumbar spine. J Electromyogr Kinesiol. 2009 Feb;19(1):39–45.

21. Lee M, Moseley A, Refshauge K. Effect of feedback on learning a vertebral joint mobilization skill. Phys Ther. 1990;70(2):97–102.

22. Petersen EJ, Thurmond SM, Buchanan SI, Chun DH, Richey AM, Nealon LP. The effect of real-time feedback on learning lumbar spine joint mobilization by entry-level doctor of physical therapy students: a randomized, controlled, crossover trial. J Manual Manipulative Ther. 9;28(4):201–11.

23. Petty NJ. The effect of posteroanterior mobilisation on sagittal mobility of the lumbar spine. Man Ther. 1995 Nov;1(1):25–9.

24. Petty NJ, Messenger N. Can the force platform be used to measure the forces applied during a PA mobilisation of the lumbar spine? J Man Manip Ther. 1996;4(2):70–6.

25. Sheaves EG, Snodgrass SJ, Rivett DA. Learning lumbar spine mobilization: the effects of frequency and self-control of feedback. J Orthop Sports Phys Ther. 2012 Feb;42(2):114–24.

26. Shum GL, Tsung BY, Lee RY. The immediate effect of posteroanterior mobilization on reducing back pain and the stiffness of the lumbar spine. Arch Phys Med Rehabil. 2013 Apr;94(4):673–9.

27. Simmonds MJ, Kumar S, Lechelt E. Use of a spinal model to quantify the forces and motion that occur during therapists’ tests of spinal motion. Phys Ther. 1995 Mar;75(3):212–22.

28. Snodgrass SJ, Rivett DA, Robertson VJ. Manual forces applied by physical therapists during cervical mobilization. J Man Manip Ther. 2006;14(3):182–3.

29. Snodgrass SJ, Rivett DA, Robertson VJ, Stojanovski E. Forces applied to the cervical spine during posteroanterior mobilization. J Manipulative Physiol Ther. 2009 Jan;32(1):72–83.

30. Snodgrass SJ, Rivett DA, Robertson VJ, Stojanovski E. A comparison of cervical spine mobilization forces applied by experienced and novice physiotherapists. J Orthop Sports Phys Ther. 2010 Jul;40(7):392–401.

31. Snodgrass SJ, Rivett DA, Robertson VJ, Stojanovski E. Cervical spine mobilisation forces applied by physiotherapy students. Physiotherapy. 2010 Jun;96(2):120–9.

32. Snodgrass SJ, Odelli RA. Objective concurrent feedback on force parameters improves performance of lumbar mobilisation, but skill retention declines rapidly. Physiotherapy. 2012 Mar;98(1):47–56.

33. Snodgrass SJ, Rivett DA, Sterling M, Vicenzino B. Dose optimization for spinal treatment effectiveness: a randomized controlled trial investigating the effects of high and low mobilization forces in patients with neck pain. J Orthop Sports Phys Ther. 2014 Mar;44(3):141–52.

34. Sran MM, Khan KM, Zhu Q, McKay HA, Oxland TR. Failure characteristics of the thoracic spine with a posteroanterior load: investigating the safety of spinal mobilization. Spine. 2004 Nov;29(21):2382–8.

35. Walsh T, Delahunt E, McCarthy Persson U. Effects of taping on thumb alignment and force application during PA mobilisations. Man Ther. 2011;16(3):264–9.

36. Zegarra-Parodi R, Pazdernik VK, Roustit M, Park PYS, Degenhardt BF. Effects of pressure applied during standardized spinal mobilizations on peripheral skin blood flow: A randomised cross-over study. Man Ther. 2016;21:220–6.
