# Supplementary material for: Spinal mobilization force-time characteristics: A scoping literature review
Source: PLoS One. 2023 Nov 14;18(11):e0289462. doi: 10.1371/journal.pone.0289462 (PMC10645339; doi:10.1371/journal.pone.0289462)
Supplement: S3 File — (DOCX) [file pone.0289462.s004.docx]

**Appendix 3: Results tables by spinal region**

**Table A: Force-time characteristics reported by studies in which spinal mobilization (SMob) was delivered to the cervical spine of humans (n=11) and inanimate objects (i.e. human analogue manikins, instrumented tools) (n=2).**

| Author(s)  Year, Country | Peak force (N)  Mean ± SD (range) | Duration (sec)  Mean ± SD (range) | Frequency (Hz)  Mean ± SD (range) | Force amplitude (N)  Mean ± SD (range) |
| --- | --- | --- | --- | --- |
| Humans | | | | |
| Conradie et al  2004, South Africa^7^ | I: (10.95-210.65)^B^ | NR | (0.28-1.00) | NR |
| Snodgrass et al  2006, Australia^28^ | C2 central; UL I: 25.6 ± 20.0; 18.8 ± 6.9 II: 40.0 ± 26.1; 29.5 ± 9.8 III: 62.5 ± 33.9; 56.4 ± 16.2 IV: 66.7 ± 27.7; 55.2 ± 13.4 C7 central; UL I: 20.8 ± 15.9; 22.1 ± 16.0 II: 36.7 ± 23.1; 34.9 ± 20.9 III: 55.9 ± 32.5; 58.1 ± 28.1 IV: 62.7 ± 38.9; 59.5 ± 36.4 | NR | C2 central; UL I: 1.00 ± 0.25; 1.03 ± 0.29 II: 0.93 ± 0.23; 0.96 ± 0.28 III: 0.90 ± 0.23; 0.87 ± 0.24 IV: 1.16 ± 0.26; 1.12 ± 0.29  C7 central; UL I: 1.08 ± 0.30; 1.05 ± 0.28 II: 0.91 ± 0.22; 0.97 ± 0.25 III: 0.92 ± 0.20; 0.86 ± 0.24  IV: 1.19 ± 0.34; 1.10 ± 0.29 | C2 central; UL I: 23.1 ± 17.8; 16.2 ± 7.6 II: 37.2 ± 23.6; 26.5 ± 9.1 III: 52.5 ± 26.3; 48.8 ± 18.2 IV: 39.5 ± 14.7; 32.6 ± 15.9 C7 central; UL I: 20.4 ± 11.4; 18.8 ± 10.1 II: 35.2 ± 15.4; 30.8 ± 17.3 III: 47.9 ± 20.2; 51.5 ± 23.8 IV: 38.1 ± 17.3; 37.2 ± 18.5 |
| Snodgrass et al  2009, Australia^29^ | C2 central:  I^R^: 22.19 (19.06-25.33^A^) II^R^: 35.05 (30.83-39.27^A^) III^R^: 56.35 (50.42-62.28^A^) IV^R^: 65.16 (58.33-72.00^A^) C2 UL:  I^R^: 22.51 (19.60-25.43^A^) II^R^: 35.05 (31.43-38.68^A^) III^R^: 56.52 (50.91-62.14^A^) IV^R^: 65.64 (59.03-72.24^A^) C7 central:  I^R^: 33.52 (29.13-37.91^A^) II^R^: 48.78 (43.55-54.02^A^) III^R^: 78.06 (70.84-85.28^A^) IV^R^: 91.80 (83.42-100.18^A^) C7 UL:  I^R^: 30.96 (27.21-34.7^A^) II^R^: 46.99 (42.16-51.83^A^) III^R^: 74.90 (68.18-81.61^A^) IV^R^: 85.29 (77.72-92.87^A^) | NR | C2 central:  I: 1.30 (1.21-1.39^A^) II: 1.18 (1.10-1.26^A^) III: 1.11 (1.02-1.19^A^) IV: 1.28 (1.19-1.37^A^) C2 UL:  I: 1.30 (1.20-1.40^A^) II: 1.19 (1.10-1.27^A^) III: 1.33 (1.23-1.43^A^) IV: 1.22 (1.15-1.29^A^) C7 central:  I: 1.32 (1.23-1.41^A^) II: 1.19 (1.11-1.27^A^) III: 1.11 (1.02-1.20^A^) IV: 1.31 (1.22-1.40^A^) C7 UL:  I: 1.29 (1.19-1.38^A^) II: 1.18 (1.10-1.27^A^) III: 1.11 (1.03-1.19^A^) IV: 1.28 (1.18-1.38^A^) | C2 central:  I^V^: 15.71 (13.38-18.04^A^) II^V^: 27.25 (23.52-30.98^A^) III^V^: 43.42 (38.37-48.47^A^) IV^V^: 35.36 (30.00-40.72^A^) C2 UL:  I^V^: 14.40 (12.51-16.29^A^) II^V^: 25.01 (22.40-27.62^A^) III^V^: 40.55 (36.37-44.73^A^)  IV^V^: 34.14 (29.71-38.58^A^) C7 central:  I^V^: 18.39 (15.61-21.17^A^) II^V^: 30.59 (27.01-34.16^A^) III^V^: 48.64 (43.28-53.99^A^) IV^V^: 38.34 (33.29-43.40^A^) C7 UL:  I^V^: 16.37 (14.01-18.73^A^) II^V^: 28.22 (25.06-31.39^A^) III^V^: 45.82 (41.05-50.59^A^)  IV^V^: 35.57 (31.09-40.05^A^) |
| Snodgrass et al  2010, Australia^30Ʊ^ | Physio; stud C2 central:  III^V^: 55.7 ± 31.5; 47.5 ± 27.2 IV^V^: 64.9 ± 36.8; 52.5 ± 26.87 C2 UL:  III^V^: 54.2 ± 28.4; 42.9 ± 23.4 IV^V^: 63.3 ± 33.3; 49.1 ± 26.5 C7 central:  III^V^: 68.8 ± 35.5; 55.6 ± 29.9 IV^V^: 80.4 ± 41.1; 63.7 ± 28.4 C7 UL:  II^V^: 40.9 ± 23.1; 34.2 ± 19.5 III^V^: 64.8 ± 31.9; 48.9 ± 25.8 IV^V^: 73.0 ± 36.2; 55.5 ± 26.2 | NR | Physio; stud C2 central:  I: 1.30 ± 0.50; 1.17 ± 0.38 II: 1.18 ± 0.46; 1.04 ± 0.35 III: 1.11 ± 0.46; 0.98 ± 0.32 C2 UL:  I: 1.30 ± 0.52; 1.17 ± 0.40 II: 1.19 ± 0.47; 1.05 ± 0.36 III: 1.12 ± 0.46; 0.99 ± 0.34 C7 central:  I: 1.32 ± 0.49; 1.18 ± 0.41 II: 1.19 ± 0.45; 1.07 ± 0.37 C7 UL:  I: 1.29 ± 0.49; 1.16 ± 0.38 II: 1.18 ± 0.45; 1.04 ± 0.33 III: 1.11 ± 0.43; 0.98 ± 0.32 | Physio; stud C2 central:  IV^V^: 35.4 ± 29.2; 28.37 ± 15.6 C2 UL:  III^V^: 40.6 ± 2.7; 33.42 ± 19.3 IV^V^: 34.14 ± 324.1; 26.8 ± 15.8 C7 UL:  III^V^: 45.8 ± 25.9; 37.49 ± 22.2 IV^V^: 35.4 ± 24.4; 29.27 ± 16.7 |
| Snodgrass et al  2010, Australia^31^ | C2 central:  I^R^: 25.42 (21.89-28.96^A^) II^R^: 32.84 (28.86-36.83^A^) III^R^: 47.82 (42.83-52.82^A^) IV^R^: 52.93 (47.98-57.89^A^) C2 UL:  I^R^: 22.97 (19.75-26.20^A^) II^R^: 30.71 (27.54-33.88^A^) III^R^: 43.62 (39.33-47.91^A^) IV^R^: 50.01 (45.14-54.88^A^) C7 central:  I^R^: 33.55 (29.86-37.24^A^) II^R^: 44.50 (40.06-48.93^A^) III^R^: 63.17 (57.15-69.19^A^) IV^R^: 72.55 (66.83-78.26^A^) C7 UL:  I^R^: 28.62 (25.68-31.56^A^) II^R^: 39.12 (35.20-43.04^A^) III^R^: 56.03 (50.74-61.32^A^) IV^R^: 64.02 (58.63-69.41^A^) | NR | C2 central:  I: 1.17 (1.10-1.27^A^) II: 1.04 (0.98-1.10^A^) III: 0.98 (0.92-1.04^A^) IV: 1.25 (1.17-1.33^A^) C2 UL:  I: 1.17 (1.10-1.24^A^) II: 1.05 (0.99-1.12^A^) III: 0.99 (0.93-1.05^A^) IV: 1.22 (1.15-1.29^A^) C7 central:  I: 1.18 (1.10-1.25^A^) II: 1.07 (1.00-1.13^A^) III: 1.00 (0.94-1.07^A^) IV: 1.24 (1.16-1.32^A^) C7 UL:  I: 1.16 (1.09-1.23^A^) II: 1.04 (0.92-1.04^A^) III: 0.98 (0.92-1.04^A^) IV: 1.21 (1.14-1.28^A^) | C2 central:  I^V^: 17.65 (14.89-20.40^A^)  II^V^: 25.40 (22.24-28.56^A^)  III^V^: 37.79 (33.61-41.98^A^) IV^V^: 28.37 (25.55-31.20^A^) C2 UL:  I^V^: 15.29 (12.90-17.67^A^) II^V^: 22.72 (20.35-25.09^A^) III^V^: 33.42 (29.93-36.91^A^) IV^V^: 26.80 (23.95-29.65^A^) C7 central:  I^V^: 19.32 (17.13-21.51^A^) II^V^: 28.79 (25.43-32.14^A^) IV^V^: 42.08 (37.27-46.89^A^) IV^V^: 32.50 (29.42-35.59^A^) C7 UL:  I^V^: 16.16 (14.32-18.00^A^) II^V^: 25.57 (22.76-28.37^A^) III^V^: 37.49 (33.48-41.50^A^) IV^V^: 29.27 (26.25-32.30^A^) |
| Gudavalli et al  2013, USA^13^ | C5; C6 Neutral traction:  DC1^TF^: 88.6 ± 9.3; 72.6 ± 17.0 DC2^TF^: 73.3 ± 14.2; 78.0 ± 14.7 DC3^TF^: 56.2 ± 19.9; 52.1 ± 29.9 DC4^TF^: 101.8 ± 8.2; 94.6 ± 23.3 Fixed flexion & traction:  DC1^TF^: 87.4 ± 12.6; 72.5 ± 16.6 DC2^TF^: 83.5 ± 21.1; 77.2 ± 21.1 DC3^TF^: 56.7 ± 35.1; 54.2 ± 27.3 DC4^TF^: 109.6 ± 19.1; 99.1 ± 22.9 Moving flexion & traction:  DC1^TF^: 101.7 ± 16.1; 90.2 ± 24.8 DC2^TF^: 127.6 ± 38.3; 119.2 ± 37.3 DC3^TF^: 84.8 ± 38.7; 79.9 ± 37.4 DC4^TF^: 107.1 ± 9.0; 91.4 ± 12.4 | NR | NR | NR |
| Snodgrass et al  2014, Australia^33^ | III, Low: 30.8 (30.7-31.0^A^) III, High: 88.6 (87.4-89.9^A^) | NR | NR | NR |
| Gudavalli et al  2015, USA^15^ | Light:  C5:13.82 ± 5.74 (0.18-23.31) Occi:17.19 ± 4.84 (0.47-26.64) Med:  C5:38.3 ± 12.12 (8.61-59.07) Occi:42.87 ± 10.40 (7.70-69.57) High:  C5:65.08 ± 17.70 (30.63-89.65) Occi:74.06 ± 16.08 (37.54-98.34) | NR | NR | NR |
| Gudavalli et al  2015, USA^16^ | No FB:  Low Ra: 12.97 ± 6.59 (0.18-26.64) Med Ra: 35.57 ± 14.87 (7.70-69.57) High Ra: 64.10 ± 17.71 (30.63-98.81) With FB:  Low Ra: 18.09 ± 1.20 (13.22-21.38) Med Ra: 44.01 ± 5.76 (16.59-49.24) High Ra: 78.96 ± 11.14 (48.27-92.21) | NR | NR | NR |
| Kope et al  2018, Canada^19^ | III, Clin1: 15.7 ± 3.3 III, Clin2: 5.6 ± 0.5 III, Clin3: 21.1 ± 2.1 III, Clin4: 16.2 ± 5.0 III, Clin5: 7.9 ± 0.3 | 60 | Clin1: 1.4  Clin2: 2.4 Clin3: 1.6 Clin4: 1.6 Clin5: 1.8 | NR |
| Chia et al  2021, Malaysia^4^ | I: 10.33 ± 0.13 II: 24.68 ± 0.01 III: 38.23 ± 0.02 IV: 45.24 ± 0.03 | NR | NR | NR |
| Inanimate objects | | | | |
| Buckingham et al  2007, Australia^3^ | 122.86 ± 50.16  (42.17-180.75) | NR | NR | NR |
| Walsh et al  2011, Ireland^35^ | III, Untaped: 70.9 ± 27.4  III, Taped: 70.3 ± 28.3 | NR | NR | NR |

Abbreviations: C: cervical, Clin: clinician, DC: doctor of chiropractic, FB: feedback, High: high force, Hz: Hertz, Light: light force, Low: low force, Med: medium force, N: Newton, NR: not reported, Occi: occiput, Physio: physiotherapist, Ra: range, SD: standard deviation, sec: seconds, Stud: students, UL: unilateral, B: grams, V: vertical direction (main line of drive), Ʊ: data only reported for significant between-group differences, A: 95% CI, R: F_resultant_, TF: traction forces.

**Table B: Force-time characteristics reported by studies in which spinal mobilization (SMob) was delivered to the thoracic spine of humans (n=2) and inanimate objects (i.e. partial cadaveric sections) (n=1).**

| Author(s)  Year, Country | Peak force (N)  Mean ± SD (range) | Duration (sec)  Mean ± SD (range) | Frequency (Hz)  Mean ± SD (range) | Force amplitude (N)  Mean ± SD (range) |
| --- | --- | --- | --- | --- |
| Humans | | | | |
| Zegarra-Parodi et al  2016, Unclear^36^ | NR | 3x60 | Non-noxious^Δ^: 28.2 (27.3-29.2^A^)  Sham^Δ^: 31.5 (30.6-32.5^A^)  Low-pressure^Δ^: 31.2 (30.2-32.2^A^) | NR |
| Funabashi et al  2021, Canada^10^ | IV, Clin-pat^R^: 323.2 ± 67.4 IV, Pat-table^R^: 296.7 ± 63.8 | NR | NR | NR |
| Inanimate objects | | | | |
| Sran et al  2004, Canada^34^ | 145 ± 38  (106-223) | NR | 0.5 | NR |

Abbreviations: Clin: clinician, Hz: Hertz, N: Newton, NR: not reported, Pat: patient, SD: standard deviation, sec: seconds, R: F_resultant_, A: 95% CI, Δ: data are reported as cycles/minute.

**Table C: Force-time characteristics reported by studies in which spinal mobilization (SMob) was delivered to the lumbopelvic spine of humans (n=17) and inanimate objects (i.e. instrumented tools) (n=1).**

| Author(s)  Year, Country | Peak force (N)  Mean ± SD (range) | Duration (sec)  Mean ± SD (range) | Frequency (Hz)  Mean ± SD (range) | Force amplitude (N)  Mean ± SD (range) |
| --- | --- | --- | --- | --- |
| Humans | | | | |
| Lee et al  1990, Australia^21^ | NR | 45 | NR | NR |
| Petty  1995, England^23^ | IV: 92.5 ± 14.0 | NR | 4.5 ± 0.5 | 9.6 ± 3.1 |
| Harms & Bader  1997, England^17^ | I^Ֆ^: 35; 42; 34 II^Ֆ^: 50; 58; 52 III^Ֆ^: 137; 135; 132 IV^Ֆ^: 158; 155; 156 (63-347) | NR | NR | NR |
| Harms et al  1999, England^18^ | G1, III: 164* G2, III: 168* III-IV: (165-190) | NR | NR | NR |
| Goodsell et al  2000, Australia^12^ | 137  (60-230) | 60 | NR | NR |
| Allison et al  2001, Australia^1^ | 146 ± 8 | 120 | 1.5*  (1.42-1.56) | NR |
| Chiradejnant et al  2001, Australia^5^ | IV^V^: 273.0 (203.6-273.0) II^V^: 161.3 (80.0-161.3) | NR | NR | NR |
| Chiradejnant et al  2002, Australia^6^ | I^V^: 64.0 ± 25.6 II^V^: 115.9 ± 43.8 III^V^: 191.3 ± 75.6 IV^V^: 227.3 ± 54.2 | NR | NR | I: 16.6 ± 7.3 II: 48.4 ± 32.7 III: 102.4 ± 61.6 IV: 32.9 ± 33.8 |
| Cook et al  2002, USA^8^ | I: 52.16 ± 36.11 (8.88-142.76)  II: 119.23 ± 50.96 (32.28-246.28) III: 179.31 ± 63.34 (64.60-307.51) IV: 242.25 ± 69.17 (117.96-425.00) | 30 | NR | NR |
| Cook  2003, USA^9^ | M; F  I: (10-125); (10-142) II: (45-154); (32-230) III: (70-275); (60-310) IV: (135-430); (120-370) | 30 | NR | NR |
| Krekoukias et al  2009, England^20^ | 103.3 ± 3.9  (96–110.2) | 120 | 1.2 | 41.1 ± 4.96  (32.3–55.6) |
| Sheaves et al  2011, Australia^25^ | Constant; Intermittent; Self-controlled  II, Pre: 25.0 (15.6-34.4^A^); 32.0 (16.6-47.3^A^); 23.1 (15.1-31.0^A^) II, Post: 13.7 (8.7-18.6^A^); 9.7 (6.4-13.0^A^); 6.7 (4.4-9.0^A^) II, FU: 24.5 (15.0-34.1^A^); 14.8 (7.1-22.5^A^); 19.3 (12.5-26.1^A^) | NR | Constant; Intermittent; Self-controlled  Pre: 0.40 (0.26-0.54^A^); 0.30 (0.19-0.40^A^); 0.29 (0.20-0.38^A^) Post: 0.22 (0.14-0.29^A^); 0.19 (0.12-0.26^A^); 0.18 (0.11-0.26^A^) FU: 0.30 (0.21-0.40^A^); 0.29 (0.19-0.39^A^); 0.24 (0.17-0.30^A^) | Constant; Intermittent; Self-controlled  Pre: 22.2 (14.6-29.8^A^); 19.5 (10.9-28.2^A^); 15.3 (9.6-20.9^A^) Post: 13.1 (8.9-17.4^A^); 8.3 (5.1-11.6^A^); 6.3 (4.2-8.4^A^) FU: 21.0 (13.3-28.7^A^); 13.3 (8.4-18.1^A^); 9.5 (5.8-13.1^A^) |
| Snodgrass & Odelli  2012, Australia^32^ | Pre; post I-IV, Sess1*: 17.4 (7.3-33.4); 7.4 (3.3-15.3) I-IV, Sess2*: 14.8 (6.1-32.4); 8.2 (3.5-17.1) I-IV, Sess3*: 10.8 (5.1-20.4); 7.0 (3.4-11.9) I-IV, FU*: 14.6 (6.6-27.4); 14.07 (6.5-35.4) | 10 | Pre; post Sess1*: 0.21 (0.09-0.36); 0.16 (0.06-0.26) Sess2*: 0.19 (0.09-0.30); 0.15 (0.07-0.27) Sess3*: 0.17 (0.07-0.29); 0.12 (0.06-0.24) FU*: 0.17 (0.08-0.30); 0.22 (0.09-0.35) | Pre; post Sess1*: 12.3 (5.6-25.7); 8.8 (3.7-16.9) Sess2*: 13.3 (6.7-27.1); 8.0 (4.0-13.8) Sess3*: 11.7 (5.0-20.7); 6.4 (3.1-12.8) FU*: 13.1 (6.6-27.5); 14.5 (5.7-27.0) |
| Shum et al  2013, England^26^ | III, Asym: 104.9 ± 34.0  III, Symp: 120.8 ± 26.8 | NR | NR | NR |
| Gudavalli & Cox  2014, USA^14^ | Nov; exper PA: 86 ± 45; 140 ± 43 Nov pre; post  PA: 86 ± 45; 102 ± 43 | NR | NR | NR |
| Gagnon et al  2016, Canada^11^ | Physios; stud L2: I: 77.0 ± 23.5; 42.6 ± 13.6 II: 82.3 ± 26.2; 43.3 ± 13.1 III: 142.6 ± 68.6; 75.5 ± 23.3 IV: 146.1 ± 56.9; 79.3 ± 26.3 L4: I: 82.2 ± 26.5; 48.9 ± 8.5 II: 91.1 ± 32.1; 54.3 ± 11.6 III: 148.3 ± 71.8; 91.6 ± 21.1 IV: 162.3 ± 64.4; 95.3 ± 25.8 | 30 | Physios; stud L2: I: 1.9 ± 0.29; 1.9 ± 0.34 II: 1.2 ± 0.44; 1.2 ± 0.17 III: 1.2 ± 0.41; 1.1 ± 0.16 IV: 2.0 ± 0.18; 2.0 ± 0.42 L4: I: 1.9 ± 0.25; 1.9 ± 0.46 II: 1.1 ± 0.41; 1.1 ± 0.10 III: 1.2 ± 0.44; 1.1 ± 0.25 IV: 2.0 ± 0.23; 2.0 ± 0.44 | Physios; stud L2: I: 31.7 ± 18.6; 17.9 ± 6.1 II: 47.4 ± 27.6; 25.1 ± 6.6 III: 74.3 ± 46.3; 38.5 ± 13.1 IV: 48.5 ± 23.4; 26.5 ± 11.9 L4: I: 32.3 ± 17.3; 23.3 ± 6.7 II: 55.5 ± 30.4; 33.8 ± 8.1 III: 82.0 ± 50.4; 53.5 ± 19.9 IV: 51.9 ± 28.3; 37.2 ± 16.1 |
| Petersen et al  2020, USA^22^ | Exper; stud  III:  Begin: 117.4 ± 19.4; 154.6 ± 57.3  End: 107.5 ± 21.1; 146.1 ± 37.9 IV: Begin: 104.7 ± 18.2; 147.4 ± 51.1  End: 94.1 ± 21.7; 144.4 ± 39.4 | NR | Exper; stud  III:  Begin: 1.4 ± 0.09; 0.97 ± 0.32;  End:1.4 ± 0.10; 1.3 ± 0.27 IV: Begin: 1.5 ± 0.11; 1.4 ± 0.53 End: 1.3 ± 0.27; 1.5 ± 0.34 | Exper; stud  III:   Begin: 1.4 ± 0.09; 0.97 ± 0.32  End: 1.4 ± 0.10; 1.3 ± 0.27 IV: Begin: 1.5 ± 0.11; 1.4 ± 0.53 End: 1.3 ± 0.27; 1.5 ± 0.34 |
| Inanimate objects | | | | |
| Björnsdóttir & Kumar  2003, Canada^2^ | Pre; Post II, Exper: 77.1 ± 25.9 (42.1-118.6); 72.1 ± 19.1 (36.3-108.5) II, Inexper: 61.1 ± 20.5 (35.8-100.2); 62.4 ± 13.0 (46.2-89.4) | 30 | NR | NR |

Abbreviations: Asym: asymptomatic, Exper: experience, F: female, Hz: Hertz, Inexper: inexperienced, L: lumbar, M: male, N: Newton, Nov: novice, NR: not reported, PA: posterior-anterior, Physios: physiotherapists, Pre: pre-intervention, Post: post-intervention, Sess: session, SD: standard deviation, sec: seconds, Stud: students, Symp: symptomatic, FU: follow-up, *: median (IQR), Ֆ: data collected over different sessions, A: 95% CI, V: vertical direction (main line of drive).

**Table D: Force-time characteristics reported by studies in which spinal mobilization (SMob) was delivered to inanimate objects (i.e. instrumented tools) with no region specified (n=2).**

| Author(s)  Year, Country | Peak force (N)  Mean ± SD (range) | Duration (sec)  Mean ± SD (range) | Frequency (Hz)  Mean ± SD (range) | Force amplitude (N)  Mean ± SD (range) |
| --- | --- | --- | --- | --- |
| Inanimate objects | | | | |
| Simmonds et al  1995, Canada^27^ | I-V, (1.74-360.54) | NR | NR | NR |
| Petty & Messenger  1996, England^24^ | Pinch-grip: (89.6-113.6) Force plate: (90.7-117.2) | 20 | Pinch-grip^Δ^: (28-32) Force plate^Δ^: (28-32) | 0.84 ± 2.3% error |

Abbreviations: Hz: Hertz, N: Newton, NR: not reported, SD: standard deviation, sec: seconds, Δ: data are reported as cycles/minute.
